# Characterization of the Zebrafish *Elastin a* (*elna^sa12235^*) Mutant: A New Model of Elastinopathy Leading to Heart Valve Defects

**DOI:** 10.3390/cells12101436

**Published:** 2023-05-21

**Authors:** Marie Hoareau, Naïma El Kholti, Romain Debret, Elise Lambert

**Affiliations:** Laboratoire de Biologie Tissulaire et Ingénierie Thérapeutique (LBTI), UMR CNRS 5305, Institut de Biologie et Chimie des Protéines, Université de Lyon 1, 7 Passage du Vercors, CEDEX 07, F-69367 Lyon, France; marie.hoareau@ibcp.fr (M.H.); elkholti@unistra.fr (N.E.K.); romain.debret@ibcp.fr (R.D.)

**Keywords:** elastin, elastinopathies, zebrafish, animal model, cardiac valves

## Abstract

Elastic fibers are extracellular macromolecules that provide resilience and elastic recoil to elastic tissues and organs in vertebrates. They are composed of an elastin core surrounded by a mantle of fibrillin-rich microfibrils and are essentially produced during a relatively short period around birth in mammals. Thus, elastic fibers have to resist many physical, chemical, and enzymatic constraints occurring throughout their lives, and their high stability can be attributed to the elastin protein. Various pathologies, called elastinopathies, are linked to an elastin deficiency, such as non-syndromic supravalvular aortic stenosis (SVAS), Williams–Beuren syndrome (WBS), and autosomal dominant cutis laxa (ADCL). To understand these diseases, as well as the aging process related to elastic fiber degradation, and to test potential therapeutic molecules in order to compensate for elastin impairments, different animal models have been proposed. Considering the many advantages of using zebrafish, we here characterize a zebrafish mutant for the *elastin a* paralog (*elna^sa12235^*) with a specific focus on the cardiovascular system and highlight premature heart valve defects at the adult stage.

## 1. Introduction

Tropoelastin is essential for the formation of elastic fibers. These elastic fibers are responsible for the elasticity of tissues in the body, such as the skin, lungs, tendons, and blood vessels [1]. The elastic component of the vascular wall is primarily needed in elastic arteries, comprising the aorta and its first ramifications. These high-caliber arteries allow the smoothing of the cardiac pulsatile blood flow into a continuous flow so that all organs are optimally perfused at all times [2,3].

In humans, mutations affecting the elastin gene have been associated with diseases such as Williams–Beuren syndrome (WBS), autosomal dominant cutis laxa (ADCL), and non-syndromic supravalvular aortic stenosis (SVAS) [4,5]. Vascular complications frequently develop and can lead to severe outcomes, namely stenosis, aneurysms, and dissections. The aorta is particularly affected; however, other large vessels, such as the pulmonary artery, can be damaged as well [6,7,8]. Moreover, these pathologies are found to increase the risk of cardiac valvulopathies [6].

Currently, there is no available treatment, neither to restore functional elastic fibers nor to prevent vascular complications that are associated with elastin deficiency. Establishing new models and an innovative research axis could uncover processes that are still not understood and open new directions. Elastin-associated pathologies have been mainly studied through the use of murine models so far. A new approach could be interesting to support the results found in mice and possibly complete them with new insights.

Zebrafish (*Danio rerio*) is a rising alternative model that has been increasingly used to study human diseases in the past few years. Their easy and low-cost maintenance, small size and numerous descendances, ex utero embryonic development, and transparency of the larvae are undeniable advantages [9,10]. In addition, their use in high-throughput assays combined with the complexity of a vertebrate species are valuable characteristics that allow faster experimental progress than with previous models [11]. Zebrafish have been used widely in various biomedical areas, including research on cardiovascular pathologies [12,13]. They have been shown to share a lot of similarities with humans, with around 82% of disease-associated human genes having at least one ortholog in the zebrafish [14]. They also show high levels of genetic and protein sequence conservation. However, due to the third-round whole genome duplication (3R WGD), a lot of their genes are present in more than one copy [15,16]. Tropoelastin is, for example, encoded by two genes in the zebrafish: *elastin a* and *elastin b* (also referred to as *elastin 1* and *2*) [17].

Recently, we described the localization of elastin in the cardiovascular system of zebrafish, particularly in the bulbus arteriosus, the atrio-ventricular, and ventriculo-bulbar cardiac valves, and in vessels [18], as it had already been shown to some extent by Miao et al. [19]. Zebrafish mutants of both elastin a and elastin b have been generated as part of the Zebrafish Mutation Project (ZMP), some of which are commercially available [20]. Nevertheless, to our knowledge, none of these mutants has yet been described.

In this article, we present and characterize a new zebrafish mutant for the *elastin a* gene (*elna^sa12235^*). Basic parameters such as survival, weight, and size were collected, and the impact of the mutation on the cardiovascular system was assessed with a particular focus on cardiac valves. This new pathological model could henceforth be used to study cardiac valve pathophysiology and to further understand the role of elastin in zebrafish cardiovascular functions.

## 2. Materials and Methods

### 2.1. Zebrafish Husbandry

All animals were kept in a dedicated facility (PRECI—UMS 3444, SFR Biosciences) with a controlled environment: 28 °C; 14 h/10 h light/dark cycle, as stated by standard procedures [21]. Mutant zebrafish were purchased from the Zebrafish International Resource Center (ZIRC) as heterozygous eggs (*elna^sa12235^*—Transcript ID: ENSDART00000102316). Since the crossing of these original eggs, our study has been conducted over four generations. All procedures were conducted in accordance with the guidelines of the European Union and French laws and approved by the local animal ethic committee under the regulatory control of governmental authority (CECCAPP, Comité d’Evaluation Commun au Centre Léon Bérard, à l’Animalerie de transit de l’ENS, au PBES et au laboratoire P4 (n° C2EA15), APAFIS #33958-2021100412005226 v6).

### 2.2. Genotyping

Zebrafish were genotyped by performing a tail biopsy on individuals 3 months post fertilization (mpf). Zebrafish were anesthetized using buffered tricaine methanesulfonate (MS-222—pH 7) at 0.2 g/L in an E3 medium. The tail biopsies were kept in 70 mM NaOH until digestion (10 min at 95 °C), then lysates were neutralized with 1 M Tris-HCl, pH 8, and centrifuged for 5 min at 10,000 g. The supernatants were collected and used for ARMS-PCR, as described in the results section, using the Taq 2X Master Mix from BioLabs (M0270). The PCR amplification products were separated by migration on a 2% agarose gel in TAE 1X at 100 V, and bands were revealed by a 20 min ethidium bromide bath followed by UV exposition.

### 2.3. Survival and Weight

After genotyping, zebrafish were separated into different tanks but fed the same way. The fish were counted regularly to note any deaths in each group and spot complications. The fish weight was measured after anesthesia by immersion using buffered tricaine methanesulfonate (MS-222—pH 7) at 0.2 g/L in an E3 medium. They were briefly wiped to remove excess water and placed on a scale. For each fish, a photo was also taken to determine its size afterward. The size was measured from the mouth of the fish to the beginning of its caudal fin to prevent bias due to variation in the total length of the tail fin. The fish were then put back in their original tanks. The body mass index (BMI) was defined as weight over size^2^ (g/cm^2^).

### 2.4. Histology

Four fish of each genotype were collected at 12 and 22 mpf and were euthanized using 400 mg/L tricaine methanesulfonate (MS-222) buffered at pH 7. Zebrafish hearts were quickly collected, fixed with formalin, embedded in paraffin (FFPE) or in Tissue-Tek^®^ O.C.T. compound, then cut into 5 or 8 µm thick sections, respectively.

#### 2.4.1. Elastic Fiber Staining—Orcein Acid (According to Shikata’s Method) 

Orcein staining was carried out on paraffin-embedded tissue sections, after dewaxing and rehydration up to water, by immersing sections for 30 min in a commercial solution of orcein acid (#010251, Diapath, Bergamo, Italy) [22,23]. The slides were then rinsed in different solutions, and counterstaining was then performed for 5 min with hematoxylin counterstain solution (#H-3401-500, Vector Laboratories, Burlingame, CA, USA). The sections were then rinsed in running tap water, dehydrated, mounted in an organic mounting medium (BioMount DPC, Biognost, Zagreb, Croatia), and scanned using the Axioscan 7 slide scanner (Zeiss, Paris, France).

#### 2.4.2. Collagen Fiber Staining—Picrosirius Red

Dewaxed and rehydrated tissue sections were stained for 1 h with Sirius Red F3B (Direct Red 80, #365548, Sigma-Aldrich, Saint Quentin-Fallavier, France) solution at 1 g/L in a saturated aqueous solution of picric acid (1.3%). After two quick washes in 0.5% acidified water, the sections were dehydrated, mounted, and scanned using the Axioscan 7 slide scanner (Zeiss) under brightfield filters.

#### 2.4.3. Lipid Staining—Oil Red O

A total of 8 µm cryosections were rehydrated for 5 min in PBS and immersed for 5 min in a 60% isopropanol solution before being stained for 15 min in a freshly prepared 0.3% (*w*/*v*) Oil Red O solution in 60% isopropanol. The stained sections were then rinsed in 60% isopropanol and counterstained using hematoxylin (#H-3401-500, Vector Laboratories) for 2 min before being mounted in an aqueous mounting medium.

#### 2.4.4. Immunohistochemistry

After deparaffinization and rehydration, the antigen retrieval step was carried out by incubating 5 µm sections (i) in pepsin reagent (#R2283, Sigma–Aldrich, Saint Quentin-Fallavier, France) for 5 min at 37 °C for elastin immunodetection or (ii) in 10 mM sodium citrate buffer, pH 6, at 98 °C for 20 min for CD3e immunolabeling. Endogenous peroxidases were then quenched for 10 min at room temperature (RT) in Bloxall^®^ Endogenous Blocking Solution (#SP-6000-100, Vector Laboratories, Burlingame, CA, USA), and non-specific sites were blocked for 30 min at RT with BlockAid™ Blocking Solution (#B10710, Invitrogen™, Illkirch-Graffenstaden, France). Elastin (#21600 (1/2000), Abcam, Waltham, MA, USA) or CD3e (#MA1-90582 (1/200), ThermoFisher, Waltham, MA, USA) primary antibodies were incubated in a blocking solution overnight at 4 °C, and biotinylated secondary antibodies were incubated for 30 min at RT. Revelation was performed using ABC Reagent and 3,3′-Diaminobenzidine (DAB) chromogen (R.T.U. Vectastain Universal Elite ABC Kit from Vector Laboratories, #PK-7200, Burlingame, CA, USA), as recommended by the manufacturer, and nuclei were counterstained using hematoxylin (#H-3401-500, Vector Laboratories).

### 2.5. Statistical Analysis

The data were analyzed for statistical significance using Prism (version 8.0.1, GraphPad Software, Boston, MA, USA). All sets containing more than 10 values per group were tested for normality with Shapiro–Wilk’s test and for homoscedasticity with Bartlett’s test. When normality and homoscedasticity were verified, parametric tests were used, and when not, non-parametric tests were run instead. Precise test choices are mentioned in the figure legend of the corresponding data. 

## 3. Results

### 3.1. The ARMS-PCR Genotyping Technique

The *elna^sa12235^* mutation consists of a single point mutation that replaces a thymine with an adenine. This change leads to the loss of a tyrosine (Tyr88) for a stop codon at the very beginning of the protein sequence. Thus, the resulting protein is truncated (only 87 amino acids) and is likely to be degraded rapidly [24,25].

**Figure 1:**
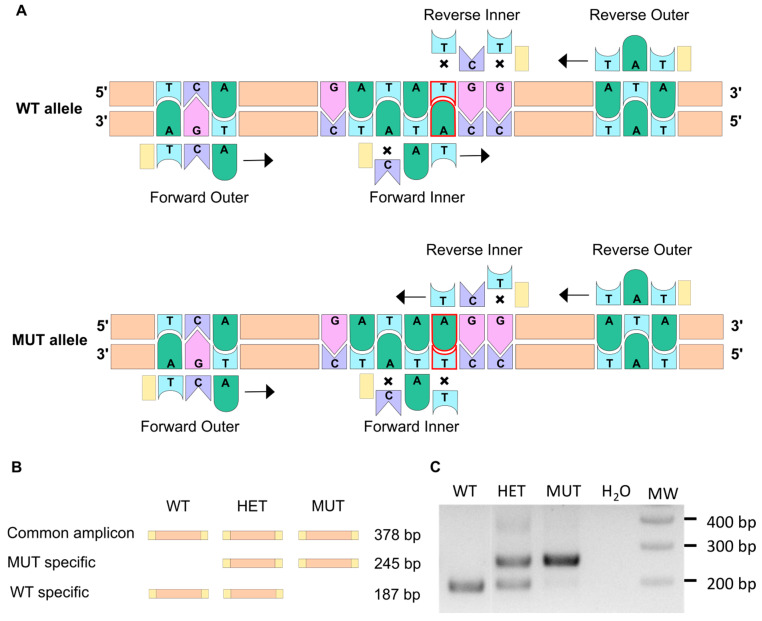
The ARMS-PCR technique allows to discriminate between WT and mutant alleles. (**A**) The ARMS-PCR principle is illustrated here. The single point mutation in the *elna^sa12235^* mutant corresponds to the substitution of thymine into an adenine (delineated in red). Two pairs of primers are used so that PCR amplification does not lead to the same result for WT and mutant alleles. Crosses symbolize a mismatch between the primer and the DNA sequence. When two mismatches are found at the 3′ extremity of the primer, the elongation cannot happen. When only one mismatch is present, transcription can occur (black arrow). (**B**) Schematic representation of amplicons generated through ARMS-PCR. (**C**) Gel electrophoresis of the PCR amplification products enables the genotype to be revealed for WT (wild type), HET (heterozygous), or MUT (mutant) individuals.

The amplification-refractory mutation system (ARMS) PCR method allows discrimination between two almost identical DNA sequences [26,27], and thus, we decided to use it for fish genotyping. This method relies on the presence of a few mismatching bases in the primer sequence (Table 1), as illustrated in Figure 1A. Indeed, it has been shown that a single mismatch is not sufficient to prevent the elongation process, whereas two mismatches at the 3′ extremity of the primer do have a significant impact [28,29]. Two pairs of primers were therefore designed using a web-based program, available at: http://primer1.soton.ac.uk/primer1.html (accessed on 10 January 2022) [30], so that each allele amplification results in a different set of amplicons with specific sizes (Figure 1B). This way, the discrimination between homozygous mutants (MUT; *elna^sa12235/sa12235^*), heterozygous mutants (HET; *elna^sa12235/+^*), and wild-type fish (WT; *elna*^+/+^) can be obtained accurately, as shown in Figure 1C. It has to be noted that through this specific PCR method, we should visualize, with the forward and reverse outer primers, an amplicon of 378 bp, as drawn in Figure 1B. However, as already mentioned by Peng and colleagues, the two pairs of primers (outer and inner primers) compete with one another in the system, and DNA polymerase typically prefers to amplify shorter amplicons, which explains why the 378 bp band is difficult to observe in Figure 1C [27]. This genotyping was also confirmed by quantitative PCR (Figure S1). Further, as expected, ARMS-PCR genotyping of adult fish from 3 clutches obtained by crossing heterozygous fish from ZIRC (*elna^sa12235/+^*) showed a Mendelian distribution (Figure S2).

### 3.2. Survival and Weight

**Figure 2:**
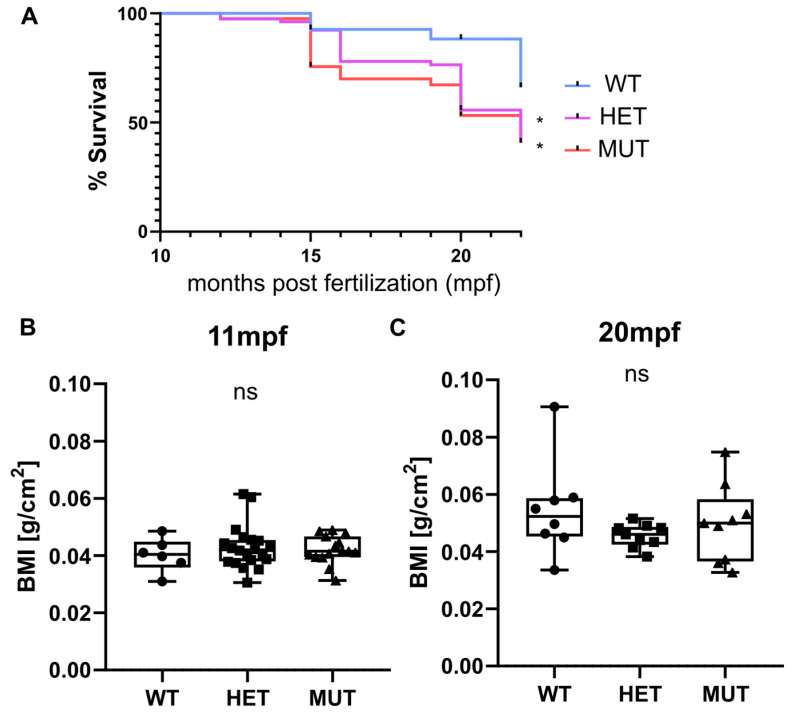
*elna* mutants have a shorter life expectancy. (**A**) Zebrafish survival from 3 independent clutches resulting from the crossing of heterozygous (*elna ^sa12235/+^*) fish was recorded after genotyping, from 10–11 mpf to 15–22 mpf (WT = 27; HET = 78; MUT = 41 zebrafish). Survival curves were compared using the log-rank Mantel-Cox test and the Log-rank test for trend; * *p* < 0.05. (**B**,**C**) Weight and size of fish were measured at 11 and 20 mpf and expressed as a body mass index (BMI). Data were analyzed by Kruskal–Wallis test; ns = non-significant; mpf = months post fertilization; WT = wild type (*elna^+/+^*); HET = heterozygous (*elna ^sa12235/+^*); MUT = mutant (*elna ^sa12235/ sa12235^*).

Survival, weight, and size were monitored for three independent clutches. Heterozygous (*elna^sa12235/+^*) and homozygous (*elna^sa12235/sa12235^*) mutants were found to have a significantly decreased life expectancy compared with their wild-type siblings. Almost half of the mutant fish were dead at 20 months post fertilization (mpf) (56% for HET and 53% for MUT), whereas only 12% of mortality was observed in the wild-type group (Figure 2A). The tracking was stopped at 22 mpf as “old” zebrafish started to die regardless of their genotype.

Zebrafish size and weight were also measured at 11 and 20 mpf, and BMI was thus calculated. Overall, there was no significant difference observed between genotypes (Figure 2B). Zebrafish globally tended to gain weight with age more than the increase in size, showing a higher BMI at 20 mpf than at 11 mpf. We can note that the populations become more heterogeneous with age independently of the zebrafish genotype (Figure 2B).

### 3.3. Severe but Highly Heterogenous Complications

Consistent with their decreased lifespan, heterozygous (*elna ^sa12235/+^*) and homozygous (*elna ^sa12235/^ ^sa12235^*) mutants were found to develop severe complications around 9–15 mpf, depending on the clutch. Nonetheless, substantial heterogeneity was observed, as some mutant fish did not show any pathological change. 

**Figure 3:**
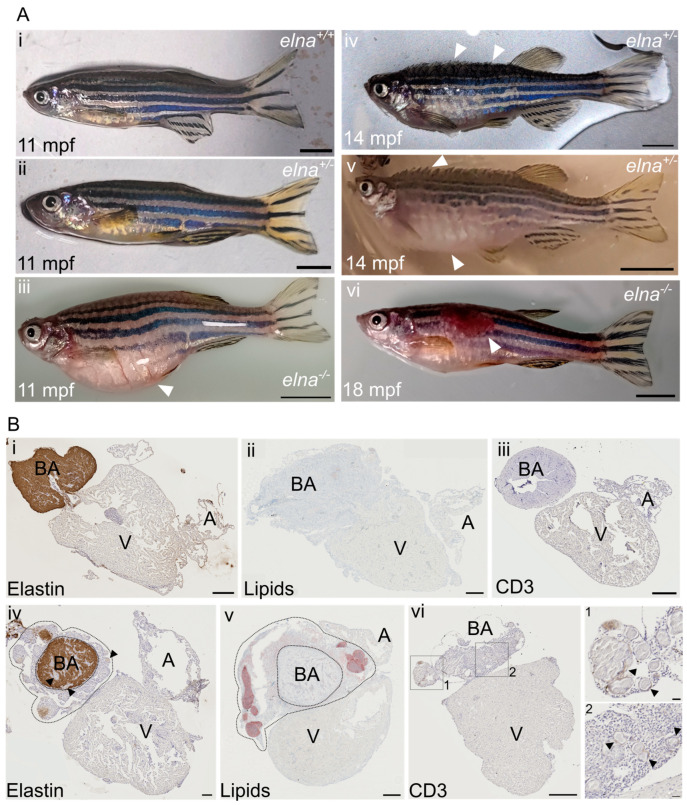
*elna^sa12235/+^* and *elna^sa12235/sa12235^* mutants suffer from severe cardiac complications. (**A**) Mutant zebrafish from 9 mpf were found to undergo complications such as (**iii**) an important swelling of the belly unrelated to stuck eggs; (**iv**,**v**) swelling of the entire body and protrusion of the scales with a rapid aggravation; (**iv**) early stage; (**v**) late stage; (**vi**) ulcers on their body; (**i**,**ii**) show normal fish of the same age. White arrows point to major observations of each image; scale bar = 0.5 cm. (**B**) Histological sections from (**i**,**iii**) normal or (**iv**–**vi**) pathological hearts from sick fish with protruding scales (here from *elna^sa12235/+^* fish) were labeled for (**i**,**iv**) elastin; (**ii**,**v**) lipids; and (**iii**,**vi**) CD3e. In (**iv**) and (**v**), dotted lines encircle the dysplastic tissue surrounding the bulbus arteriosus; black arrows point to “nodule-like” structures found in the pathological hearts; scale bar = 200 µm, except for zooms in vi-1 and vi-2, where scale bar = 20 µm. BA = bulbus arteriosus; V = ventricle; A = atrium; mpf = months post fertilization.

The most frequent complications encountered were: a swollen belly that was not due to stuck eggs in the abdomen (Figure 3(Aiii)), important ulcers (tail, trunk, and head area) (Figure 3(Avi)), and more impressively, some fish were found all swollen with protruding scales and having trouble swimming, a condition sometimes referred to as “dropsy” (Figure 3(Aiv,Av)).

When reaching this humane endpoint, the fish were euthanized and their hearts collected. During necropsy, we noticed that these hearts were bigger and surrounded by undetermined friable yellowish tissue. Histological analysis revealed pathological dysplastic tissue surrounding the bulbus arteriosus (BA). The BA was still easily distinguishable because of its elastin content, as elastin was absent in the dysplastic tissue (Figure 3(Bi,Biv)). The pathological tissue was composed of small cells with a high nuclear-to-cytoplasmic ratio and sprinkled with “nodule-like” structures, sometimes also found in the BA and the ventricle (Figure 3(Biv–Bvi)). These histological transformations were observed frequently in old fish hearts (22 mpf), regardless of the genotype (Figure S3). Further immunohistochemical and histological analyses of the heart sections revealed that the nodule-like structures corresponded to encapsulated neutral lipid-positive aggregates (Figure 3(Bv)) surrounded by inflammatory cells, as demonstrated by the T cell marker CD3e labeling (Figure 3(Bvi)).

Considering the cardiovascular macroscopic and microscopic defects observed in the hearts of *elna ^sa12235/+^* and *elna ^sa12235/^^sa12235^* fish at the adult stage, we monitored embryonic development. *elna^sa12235^* embryo survival was not affected during the early stages of development of individuals (from 0 to 7 dpf), and we did not notice any abnormality during elastin a mutant development based on daily observations. Cardiovascular dysfunction is usually associated with the formation of edema in zebrafish embryos [12]. Therefore, larval heartbeats were recorded at 7 days post fertilization, but this measurement revealed no difference, suggesting that the cardiovascular defects appear later at larval or adult stages (Figure S4).

### 3.4. Age-Associated Cardiomegaly

The hearts of 12 mpf and 22 mpf apparently healthy zebrafish were collected to determine if any difference could be observed in mutants prior to developing the complications shown in Figure 3.

**Figure 4:**
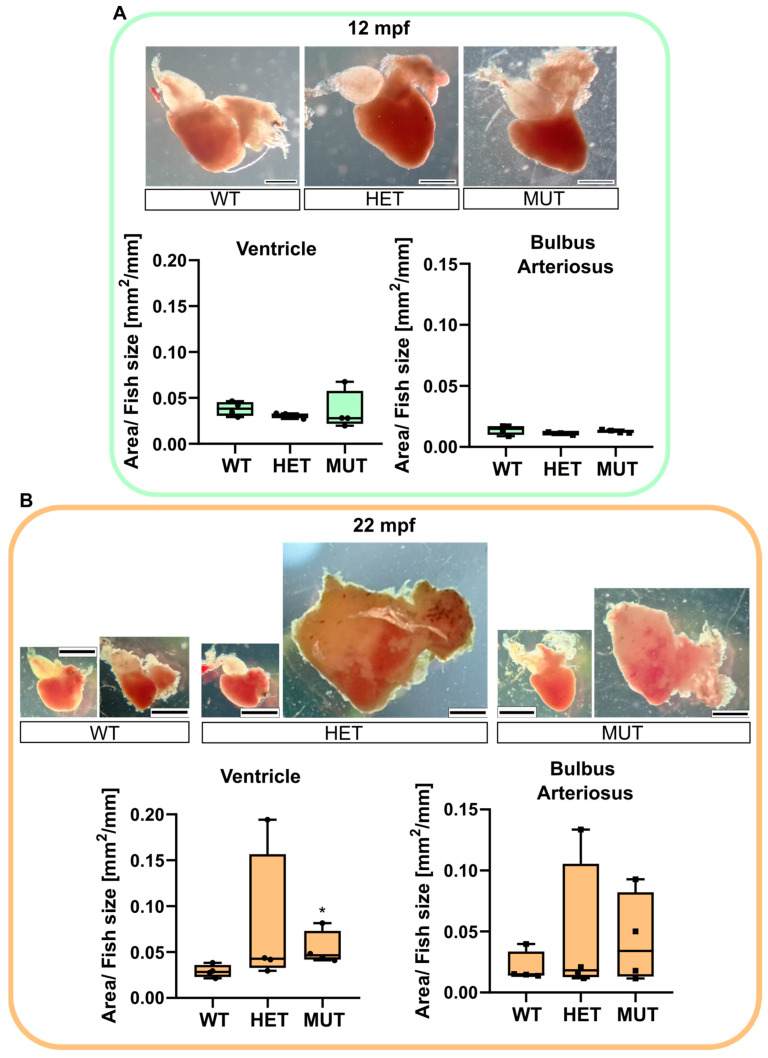
*elna* mutants are subject to cardiomegaly with age. Zebrafish hearts were collected from apparently healthy fish at 12 (**A**) or 22 (**B**) mpf (n = 4 fish per genotype and age). Representative images are shown for each genotype. The area of the ventricle and the bulbus arteriosus (including dysplastic tissue) was measured in each heart and reported, normalized with the size of the fish. Data were analyzed by Kruskal–Wallis with Dunn’s multiple comparison test; * *p* < 0.05. Scale bar = 0.5 mm. mpf = months post fertilization; WT = wild type (*elna^+/+^*); HET = heterozygous (*elna^sa12235/+^*); MUT = mutant (*elna^sa12235/sa12235^*).

No visible change was noticed comparing the 12 mpf hearts (Figure 4A). A fat layer was found surrounding the chambers of the hearts of almost all 22 mpf fish. However, the most impressive changes were found in mutant fish *(elna^sa12235/+^* and *elna^sa12235/sa12235^*), with some of their hearts being 4 to 5 times bigger than the normal size. The surface area of the BA (including the dysplastic tissue) and the ventricle were measured, and their ratio over the size of the fish was compared between genotypes (Figure 4B). *elna^sa12235/sa12235^* zebrafish had significantly bigger ventricles compared with their WT siblings. The BA of elastin mutant fish also tended to increase in size, but not significantly, probably because of the heterogeneity of the dysplastic tissue.

### 3.5. Cardiac Valve Morphology and Composition

The hearts of 12 mpf zebrafish were further analyzed by staining or immunolabelling of histological sections. Although the three chambers (BA, V, and A) did not show any obvious modifications, the cardiac valves did. 

**Figure 5:**
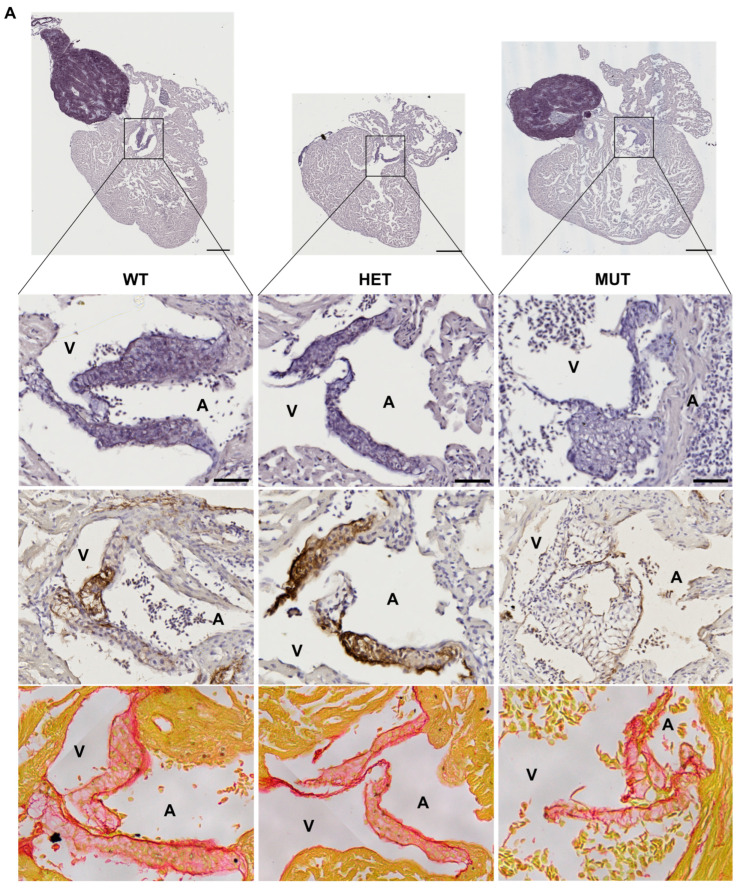

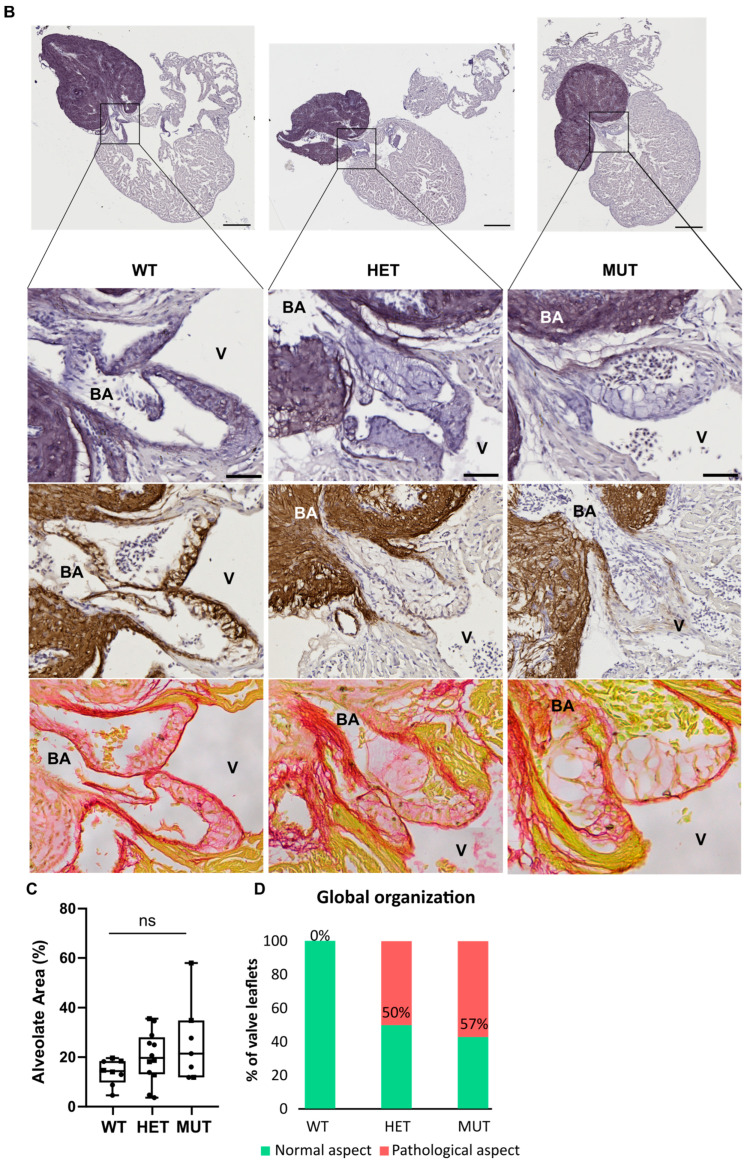
*elna* mutants show abnormal cardiac valves. (**A**,**B**) Histological images of the complete heart sections (orcein) and a zoom of the A-V valve (**A**) or BA-V valve (**B**) from 12 months post fertilization fish stained with orcein (first row), immunolabeled with anti-elastin antibodies (second row), or stained with picrosirius red (third row). (**C**) Quantification of “honeycomb-like” structures referred to as alveolate areas in valves from histological sections of zebrafish hearts. Each point is the result of one leaflet of the valve. Circles are for A-V valves, and squares are for V-BA valves. Four fish per genotype were analyzed; however, all their valves were not always visible enough to perform the analysis. (**D**) Global aspect of each valve leaflet was categorized as normal or pathological when the shape was unusual and the alveolate area exceeded 20% of the total surface. Scale bar = 200µm for complete heart sections and 40µm for valve images; A = atrium; V = ventricle; BA = bulbus arteriosus; SV = sinus venosus; A-V = atrio-ventricular; V-BA = ventriculo-bulbar; WT = wild type (*elna*^+/+^); HET = heterozygous (*elna^sa12235/+^*); MUT = mutant (*elna^sa12235/sa12235^*); Kruskall–Wallis test was performed followed by Dunnett’s post hoc test in (**C**); ns = not significant.

Mutant valves had excessively honeycomb-like areas and showed a disorganized aspect, both visible in the atrio-ventricular (A-V) (Figure 5A) and the ventriculo-bulbar (V-BA) valves (also called bulbo-ventricular valves) (Figure 5B). Moreover, they seemed larger and less defined. The width of valve leaflets is hard to measure, as it depends mostly on where the section is made. Thus, we decided to quantify the “empty” area in the honeycomb-like structures revealed by the elastin staining (by orcein) to assess the magnitude of the change. We found that these alveolate structures were also found in wild-type zebrafish valves but represented at most 20% of the total surface, whereas they could reach almost 60% in mutants (Figure 5C). Moreover, analysis of the global valve organization clearly revealed the abnormal state of the valves in more than 50% of *elna sa12235* mutants, whereas no defects were observed in the valves of WT animals (Figure 5D).

The low number of individuals in the analysis did not allow for any statistical significance to be established. Moreover, the heterogeneity of the mutant population as well as the effects of an elastin a deficiency are to be kept in mind. While many mutants appear still healthy, *elna^sa12235/+^* and *elna^sa12235/sa12235^* zebrafish are the only ones to develop pathological valves, potentially initiating changes that will evolve into the severe complications shown in Figure 3.

As might be expected, the valves of *elna^sa12235/+^* and *elna^sa12235/sa12235^* mutants were found to have a markedly diminished quantity of elastin, according to both the orcein staining and the anti-elastin immunohistochemistry assay, and this reduction seemed to be dependent on fish genotype (Figure 5). However, some signals were still present in the valves of homozygous mutants. The methods used for elastin detection are not specific to elastin a. Therefore, elastin b is probably responsible for the remaining signals.

Cardiac valves of zebrafish reaching a humane endpoint (Figure 3) were more complicated to analyze, as often the entire heart was swollen and the corresponding tissue disintegrated during the inclusion steps, making the identification of the valves complex. From all the specimens collected, only one valve could be clearly seen, and it was highly honeycombed (data not shown). Furthermore, it should be noted that most valves found in pathological hearts from 22 mpf zebrafish of all genotypes were abnormally large and sometimes honeycombed as well (Figure S3).

## 4. Discussion

The mature heart valves are highly organized connective tissue structures populated with dynamic cell populations [31]. The extracellular matrix of the valves is stratified into elastin-, proteoglycan-, and collagen-rich layers that confer distinct biomechanical properties to the leaflets and supporting structures [32].

In this article, we describe for the first time the complications resulting from elastin deficiency in adult zebrafish, specifically concerning the cardiovascular system. Both heterozygous and homozygous mutants for the *elastin a* gene have a shorter life expectancy compared to wild-type zebrafish. They are more likely to develop ulcers, general swelling of their bodies, and protruding scales, leading invariably to premature death if the experiment is not stopped. This severe phenotype seems to be mainly due to impairment in the heart, particularly in cardiac valve function. Indeed, apart from a decreased content of elastin and before the onset of pathological outcomes, the valves of *elastin a* mutants showed an abnormal aspect resembling a honeycomb pattern.

In humans, cardiac valve insufficiency is often associated with swelling of the abdomen, ankles, and feet, also called edema [33,34]. Thus, the swelling of the fish or the “dropsy” state described in this article could be equivalent to a late stage of this condition with widespread edema. Cardiac regurgitation or aortic valve stenosis, which are associated with defective valves, are also known to cause cardiac hypertrophy in humans, particularly of the left ventricle [35]. This could be similar to what happens in some mutants, explaining the impressive increase in the volume/size of their hearts.

In sick mutant zebrafish, we also described pathological tissue surrounding the bulbus arteriosus—nicely delimited by elastin immunodetection—of the heart. This abnormal tissue consisted of lipids encapsulated in nodule-like structures surrounded by inflammatory cells. Inflammatory tissues have already been described in the heart of zebrafish in the context of overload-induced cardiac hypertrophy. Cardiomyocytes are known to secrete pro-inflammatory cytokines when stressed [36]. In our case, the origin of the pathological tissue in old zebrafish and younger mutants is not known yet, and we did not find similar observations in the literature.

We can hypothesize that it is a consequence of cardiac fatigue, triggered by valve dysfunction in mutants and by global aging in older fish. Husbandry parameters can influence the development of endocardiosis and myxomatous degeneration of the cardiac valves, along with aging [37]. However, regular analyses of the fish in our animal facility are carried out by an external veterinary laboratory using PCR and histological stainings (hematoxylin and eosin staining and specific staining (GRAM, Giemsa, and Fite-Faraco)) on young and old animals from different racks, and no bacterial infection has been detected in the last five years. Furthermore, all fish were exposed to the same environment, and mutants for *elastin a* did show severe complications more than one year before their wild-type siblings. Thus, the mutation of elastin a somehow accelerates cardiovascular aging or makes mutant zebrafish more susceptible to infection.

Our study demonstrates that the mutation does not affect all carriers in the same way, suggesting an incomplete penetrance. This phenotype variability could emerge from behavioral differences during growth, giving an advantage to individuals in the group in terms of access to food, for example. This observation could correspond to the interindividual variability observed in humans with a mutated *elastin* gene, where all patients will not develop the same complications [6]. Indeed, in the skin of cutis laxa patients, elastic tissue varies in content, appearance, proportion, and the way in which elastin and microfibrillar components are associated [38]. Moreover, in humans, high variability in phenotype is also observed between members of the same family carrying the same mutation on the elastin gene, again reflecting the differential penetrance of the mutation from one individual to another [39]. Finally, this heterogeneity in phenotype might be a common feature of connective tissue disorders since it is also observed in osteogenesis imperfecta (OI) and selected mutations in *col1a1* and *col1a2* paralogs in the zebrafish model give rise to phenotypic variability, mirroring the clinical variability associated with human disease pathology [40]. Consistent with our finding, *Eln^+/−^* mice were also described as developing the cardiac valvular disease at a relatively low frequency in young mice (17%), whereas old mice were more affected, with 70% showing valve defects [41].

The heterogeneity of observed phenotypes in *elna^sa12235^* mutants can also be attributed to the strong regenerative capacity of this model. Indeed, zebrafish are able to perfectly restore injured organs, including the heart and its valves [42,43,44]. This process could intervene in the development of cardiovascular issues associated with elastin deficiency. Therefore, we can assume that larger defects in homozygous fish lead to an earlier initiation of the regeneration process in these individuals, which consequently decreases the difference in phenotype between homozygous and heterozygous animals, thus explaining our results. In addition, this remarkable regenerative competence probably has an impact on *elna^sa12235^* mutants and their survival because, despite the severe phenotype observed, particularly in the general morphology and size of the heart, no massive fibrosis is highlighted by picrosirius red staining (Figure 5 and Figure S3) as could have been observed in mammals after infarction [45,46].

As zebrafish *elna^sa12235^* mutants demonstrate a relatively severe phenotype, this model could be a valuable alternative to mice to test new therapeutic molecules. The use of elastin-based materials or the reinduction of elastin fiber synthesis in aged patients or elastin-deficient patients is a crucial challenge, particularly when the cardiovascular system is impacted, and could be alternatives to heart valve surgical repair [47,48]. Various drugs and ingredients have been investigated for their capacity to induce elastin neosynthesis, among them minoxidil, an ATP-dependent K+ (K_ATP_) channel opener, or dill extract, an activator of LOXL1 expression [49,50,51,52,53,54]. To date, various murine models have been employed to test those molecules [49,50,55,56].

*Eln^−/−^* mice have normal cardiovascular development in utero up to an embryonic day (E) 18, but die just a few days later, between day (P) 0 and P4.5, due to the inward proliferation of medial smooth muscle cells resulting in obliteration of the large vessel lumen [2,57]. On the contrary, mice haploinsufficient for elastin (*Eln^+/−^*) live a normal life span despite significant hypertension. However, since the generation of this mutated line, with repeated backcrossing to C57BL/6 mice, the blood pressure phenotype has become less severe (∼15% increase in systolic blood pressure over WT compared to the initially reported 36% increase) [58]. Thus, further analyses are necessary to highlight cardiovascular defects in those mice (i.e., *Eln^+/−^* arteries have smaller diameters and thinner walls than WT arteries and present an elevated number of elastic lamellae in the medial layer (11 ± 0.1 instead of 8.8 ± 0.4 lamellae in 6 month-old mice)) [59,60,61], which leads to a more tricky validation of the efficacy of a therapeutic molecule.

The fact that elastin exists in two copies in the zebrafish could be interesting in this particular case, as *elastin b* is still expressed in the vessels and in the bulbus arteriosus (as demonstrated by elastin immunodetection in Figure 3B and Figure S3), so that the zebrafish survives, but at the same time valves are mostly deprived of it, as elastin a seems to be the prevalent isoform expressed in this specific structure.

Another advantage of zebrafish as an animal model to validate drug efficiency is the ease of administration of a water-soluble compound. Indeed, the fish can be directly immersed in the drug solution rather than being injected into the blood circulation.

Finally, the cardiac electrophysiology (heart rate and electrocardiogram profile) of the zebrafish heart is much closer to that of humans than that of mice [11,62]. For example, the zebrafish heart contracts at a frequency of 60–100 beats/min, as in humans, whereas this rate is almost 10 times higher in mice. Considering these parameters, we can also assume that the behavior of a therapeutic molecule in zebrafish could better predict its behavior in humans compared to mice.

All these arguments lead us to consider the zebrafish model and, in particular, the *elna^sa12235^* mutants as an alternative to the traditionally used mammalian model and as an interesting tool for developing new candidates for the treatment of valvulopathies related to elastin deficiency. Obviously, given the high heterogeneity of the observed phenotype, the number of fish used to test pharmacological molecules will have to be substantial, as for the mouse model. Despite the numerous advantages offered by the zebrafish model, it will not supplant mammalian models but could significantly reduce their number and accelerate the introduction of new therapeutic molecules.

## Figures and Tables

**Table 1:** The presence of a few mismatching bases in the primer sequence.

	Primer Sequence	Tm
Forward outer	AATAATTCTCAATGGAGCCTAACTCC**TCA**	65
Reverse outer	TCCTAGAGGGAAACATGAATGCTCA**TAT**	65
Forward inner	ACATATCTCTCTGGCTGTAGGTGGA**CAT**	65
Reverse inner	CACCACCATATCCACCATAACCC**TCT**	66

## Data Availability

All relevant data are within the manuscript.

## Supplementary Materials

The following supporting information can be downloaded at: https://www.mdpi.com/article/10.3390/cells12101436/s1, Figure S1: Genotyping of WT, *elna^sa12235/+^* and *elna^sa12235/sa12235^* animals through quantitative PCR; Figure S2: Genotyping of 3 different clutches obtained from crossing heterozygote animals through ARMS-PCR; Figure S3: 22 mpf fish all show pathological hearts regardless of their genotype; Figure S4: Heart rate is not modified in *elna* mutant larvae.
