# Supplementary material for: Characterization of the Zebrafish Elastin a (elnasa12235) Mutant: A New Model of Elastinopathy Leading to Heart Valve Defects
Source: Cells. 2023 May 21;12(10):1436. doi: 10.3390/cells12101436 (PMC10217568; doi:10.3390/cells12101436)
Supplement: Supplementary file 1 [file cells-12-01436-s001.zip › cells-2320074-supplementary.pdf]

Supplementary Materials and Methods

Genotyping by qPCR

A part of the caudal tail was collected as already described and 2 distinct qPCR were performed using the following primers: Forward primer without mutation 5′-CCATATCCACCATAACCCCCA-3′, Forward primer with mutation 5′-CCATATCCACCATAACCCCCT-3′ and Reverse primer 5′-CTTCCCTCCCTGTCCTGTTG-3′. FastStart Universal SYBR Green Master (Merck) was used for qPCR reaction in a RotorGene HRM (Qiagen).

Heart rate count

Zebrafish larvae at 7 dpf were immobilized in a E3 medium drop and left undisturbed for 15 min prior to recording so that the stress of manipulation would not impact the measure unduly. Videos of the heart region were then recorded using a camera Dino-Eye Digital Eye Piece Camera (AM7025X) fixated on a binocular magnifier Olympus SZX10. 40 seconds to 1 min were recorded for each larva so that 30 sec without the fish moving could be used for analysis. Heart rate was assessed by a semi-automatic treatment on imageJ. Briefly, 30 seconds were selected from the movie, converted into tiff images, then stacked and reoriented so that the fish stays in the same place during the whole period. The heart region was then selected and a "dynamic reslice" was performed on a selected line on the stack to obtain an accumulation of this line in all the images (10 images/second). A periodic movement could then be characterized using a Fourier transform (FFT function on imageJ). The second harmonic was then highlighted (r) and multiplied by 1/300 x2 to obtain beats per minute (bpm). This method was first verified by counting manually the beats during 30 seconds put on 0.5x speed for 5 zebrafish.

Supplementary Figures

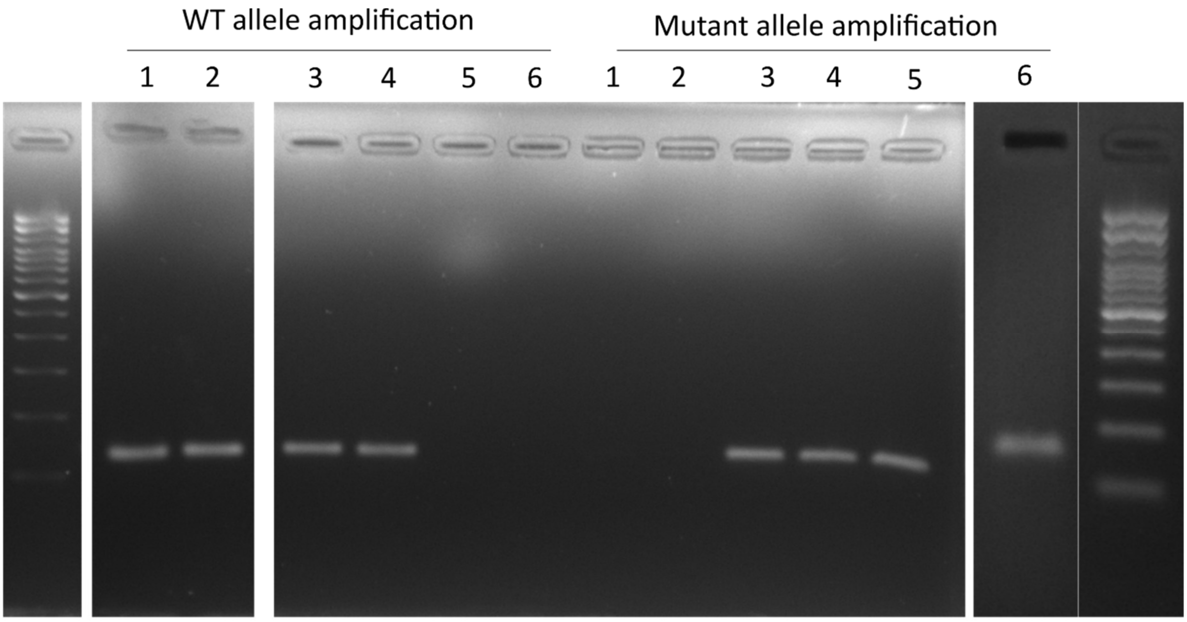

WT *elna* allele

Sample ID	Ct
1	18.07
2	18.20
3	19.18
4	19.08
5	-
6	-
water	-

Mutant *elna* allele

Sample ID	Ct
1	27.72
2	27.25
3	18.26
4	18.84
5	17.17
6	19.74
water	-

Sample ID	Genotype
1	**+/+**
2	**+/+**
3	**+/-**
4	**+/-**
5	**-/-**
6	**-/-**

Figure S1. Genotyping of WT, *elna*$^{sa12235/+}$ and *elna*$^{sa12235/sa12235}$ animals through quantitative PCR.

Number and % of individuals of each genotype at adult stage

	WT		HET		HOM		
	Nb	%	Nb	%	Nb	%	Total Nb of individuals
Clutch 1	9	21	23	52	12	27	44
Clutch 2	12	19	37	58	15	23	64
Clutch 3	6	16	18	47	14	37	38
Total	27	19	78	53	41	28	146

Figure S2. Genotyping of 3 different clutches obtained from crossing of heterozygote animals through ARMS-PCR.

A part of the caudal tail was collected and used for the genotyping of the animals through ARMS-PCR. WT = wild type (*elna*$^{+/+}$) ; HET = heterozygous (*elna*$^{sa12235/+}$) ; MUT = mutant (*elna*$^{sa12235/sa12235}$).

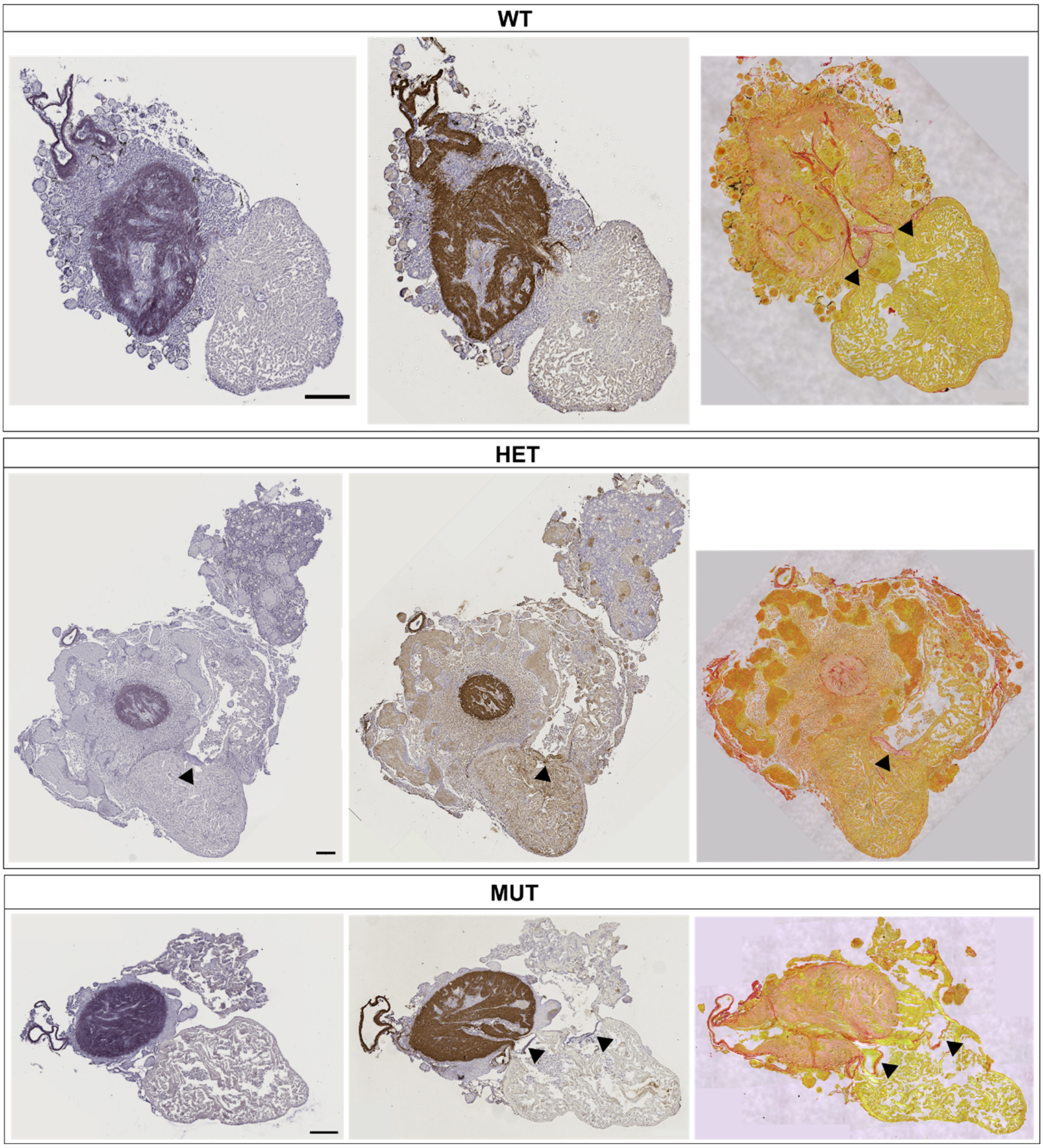

Figure S3. 22 mpf fish all show pathological hearts regardless of their genotype.

Heart sections from 22 months post fertilization (mpf) were stained with orcein (left), immunolabelled with anti-elastin antibodies (middle), or stained with picrosirius red (right). Scale bar = 200 μm. Black arrows point to visible cardiac valves. WT = wild type (*elna*$^{+/+}$) ; HET = heterozygous (*elna*$^{sa12235/+}$) ; MUT = mutant (*elna*$^{sa12235/sa12235}$).

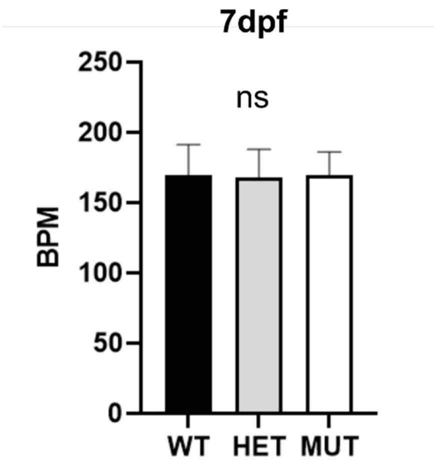

Figure S4. Heart rate is not modified in *elna* mutant larvae.

The heart rate of zebrafish larvae at 7 days post fertilization (dpf) was measured without anesthesia and reported according to their genotype. 10 to 15 larvae were assessed per genotype per experiment, and 3 experiments were repeated with different clutches. ns = non-significant, normality and homoscedasticity were tested by the Shapiro-Wilk test and the Bartlett test respectively and a one-way ANOVA was then performed followed by Dunnett's post hoc test. WT = wild type ($elna^{+/+}$) ; HET = heterozygous ($elna^{sa12235/+}$) ; MUT = mutant ($elna^{sa12235/sa12235}$).
